# Adaptive Low-Rank Tensor Estimation Model Based Multichannel Weak Fault Detection for Bearings

**DOI:** 10.3390/s24123762

**Published:** 2024-06-09

**Authors:** Huiming Jiang, Yue Wu, Jing Yuan, Qian Zhao, Jin Chen

**Affiliations:** 1 School of Mechanical Engineering, University of Shanghai for Science and Technology, Shanghai 200093, China; 223341580@st.usst.edu.cn (Y.W.); yuanjing@usst.edu.cn (J.Y.); qzhao@usst.edu.cn (Q.Z.); 2 State Key Laboratory of Mechanical System and Vibration, Shanghai Jiao Tong University, Shanghai 200240, China; jinchen@sjtu.edu.cn

**Keywords:** fault diagnosis, fault characteristics extraction, low rank, multichannel signals, rolling bearings

## Abstract

Multichannel signals contain an abundance of fault characteristic information on equipment and show greater potential for weak fault characteristics extraction and early fault detection. However, how to effectively utilize the advantages of multichannel signals with their information richness while eliminating interference components caused by strong background noise and information redundancy to achieve accurate extraction of fault characteristics is still challenging for mechanical fault diagnosis based on multichannel signals. To address this issue, an effective weak fault detection framework for multichannel signals is proposed in this paper. Firstly, the advantages of a tensor on characterizing fault information were displayed, and the low-rank property of multichannel fault signals in a tensor domain is revealed through tensor singular value decomposition. Secondly, to tackle weak fault characteristics extraction from multichannel signals under strong background noise, an adaptive threshold function is introduced, and an adaptive low-rank tensor estimation model is constructed. Thirdly, to further improve the accurate estimation of weak fault characteristics from multichannel signals, a new sparsity metric-oriented parameter optimization strategy is provided for the adaptive low-rank tensor estimation model. Finally, an effective multichannel weak fault detection framework is formed for rolling bearings. Multichannel data from the repeatable simulation, the publicly available XJTU-SY whole lifetime datasets and an accelerated fatigue test of rolling bearings are used to validate the effectiveness and practicality of the proposed method. Excellent results are obtained in multichannel weak fault detection with strong background noise, especially for early fault detection.

## 1. Introduction

With the enhancement of rotating equipment in complexity and reliability, prognostics and health management of equipment attract widespread attention. As a crucial component of rotating equipment, rolling bearings can cause the entire equipment to shut down and even lead to serious safety accidents once they fail. Therefore, “timely” bearing weak fault detection is quite important for the safe operation of high-end mechanical equipment. The vibration signal contains abundant operation status information on mechanical equipment, and early fault detection based on the vibration signal has been widely researched [1,2,3,4].

Early weak faults are usually represented as tiny changes in vibration signals, which tend to be blended in a large amount of strong noise interference, making it difficult to accurately extract and recognize. Therefore, to address these problems, some high-efficiency signal processing techniques and advanced characteristics extraction methods have been adopted to overcome signal weakness and strong background noise interference. Wang et al. proposed different low-rank and sparse estimation models for weak fault characteristics extraction of bearings under different conditions [5,6,7]. Zhao et al. [8] developed a high-concentration TFA technique, termed frequency-chirprate synchrosqueezing-based scaling chirplet transform (FCSSCT) for analyzing nonstationary and close-spaced fault frequencies of wind turbines. Li et al. [9] proposed a novel time-frequency ridge estimation (TFRE) method for extracting weak fault characteristics of bearings under variable speed conditions. Kumar et al. [10] proposed a dynamic degradation monitoring method based on variational mode decomposition (VMD) and based on trigonometric entropy measurements for early fault detection of rolling bearings. Zhang et al. [11] proposed a fast nonlinear blind deconvolution algorithm for early fault diagnosis of rotating machinery. Bin et al. [12] proposed a method combining wavelet packet decomposition (WPD) and empirical mode decomposition (EMD) for rotating machinery early fault diagnosis. Li et al. [13] proposed a new method for extracting the bearing fault characteristic frequency based on improved singular value decomposition (ISVD). Pan et al. [14] proposed a newly intelligent diagnosis method based on a semi-supervised matrixized graph embedding machine (SMGEM), which can use a few labeled samples to obtain better identification accuracy. Zhao et al. [15] proposed a novel frequency matching demodulation transform (FMDT) technique extending the generalized demodulation transform for bearing weak fault feature extraction and diagnosis under variable speeds. However, current signal processing and characteristics extraction methods for early weak faults mainly focus on dealing with single-channel signals.

With the popularity of multichannel/multisensory in the Industry 4.0 era, multichannel signals, which contain an abundance of condition information on equipment, show greater potential for weak fault characteristics extraction and early fault detection. Li et al. [16] studied composite fault diagnosis based on multi-source signals, and the compression sensing technique was utilized to process signals. Wu et al. [17] proposed a new deep long-short-term memory model to fuse multisensory monitoring signals and improve the prediction accuracy. Long et al. [18] researched the multi-sensor signals processing method with an attention mechanism and improved AdaBoost for motor fault diagnosis. In addition, to solve the multichannel and multidimensional signal processing problem, multidimensional signal processing technology has also been further developed. Multivariate empirical mode decomposition (MEMD) can realize synchronous processing and adaptive decomposition of multichannel signals [19]. Lv et al. [20] applied MEMD to synchronously analyze multivariate signals for bearing fault diagnosis. Similarly, Rehman et al. [21] extended the variational mode decomposition algorithm and proposed multivariate variational mode decomposition (MVMD) to process multivariate or multichannel data. Song et al. [22] studied the multichannel mode extraction based on MVMD and self-adaptive MVMD to realize the multichannel fault diagnosis. Zhang et al. [23] proposed a novel weighted sparsity index based on multichannel fused graph spectra for machine health monitoring. Lang et al. [24] proposed the direct MITD (DMITD) algorithm for the adaptive processing of multi-loop data, which outperforms traditional techniques in capturing both the regularity and evolution of the plant-wide oscillation from noisy signals in the nonlinear and nonstationary process. Yan et al. [25] proposed a new approach based on multivariate singular spectrum decomposition (MSSD) and improved Kolmogorov complexity (IKC), which demonstrates good performance in extracting fault information and health condition identification. In fact, in addition to containing more fault characteristic information, the potential structural information between each channel can effectively assist in weak fault characteristics extraction and fault diagnosis [26]. However, the current high-dimensional signal processing methods tend to preprocess data into matrix or vector form. This preprocessing destroys the potential structural information between channels for multichannel data, weakening the inherent advantages of multichannel signals in characteristics extraction and fault diagnosis. Thus, how to effectively utilize the advantages of multichannel signals with their information richness and explore the correlation features between multichannel structural information and fault characteristics synchronously has become the key to achieving effective fault characteristics extraction for weak fault detection.

Tensors, as a natural and direct form of data representation for high-dimensional data, can maximize the preservation of data information and structure [27]. Recently, tensor and tensor decomposition-related research work has been widely carried out in various fields and has broad application prospects in pattern recognition [28], speech processing, computer vision [29] and fault diagnosis. The structure correlation between multichannel signals and the fault modes has a certain mapping relationship, which can assist in the fault characteristics extraction from multichannel signals effectively. Based on this assumption, the multichannel processing method based on tensor decomposition has been widely researched in fault diagnosis. Hu et al. [30] proposed a tensor-based method to realize the fault diagnosis of rotating equipment. To utilize multisensory signals for the gear fault diagnosis, Cheng et al. [31] proposed a nearest neighbor convex hull tensor classifier. Yuan et al. [32] proposed a novel multichannel signal denoising method based on a high-order singular value decomposition. The multichannel signal processing methods based on tensor decomposition can fully utilize the topological structure and correlation between different channels and more effectively extract the structural correlation features from high-dimensional datasets, which has been a new way for weak fault characteristics extraction of multichannel signals.

Tensor singular value decomposition (TSVD), as one of the tensor decomposition methods, has a promising application in signal characteristics extraction under multichannel conditions. Zeng et al. [33] proposed a multispectral image denoising method based on TSVD and tensor nuclear norm (TNN). Liu et al. [34] presented a tensor train-TSVD algorithm for data reduction. Song et al. [35] proposed a transformed TSVD-based method for tensor completion. TSVD-based methods have achieved excellent applications in image denoising and data recovery, and TSVD is theoretically fully applicable to the processing of mechanical fault signals. Ge et al. [36] studied tensor robust principal component analysis (TRPCA) based on TSVD and achieved good applications in bearing fault diagnosis. Therefore, the TSVD-related method provides a promising way for multichannel signal processing in the tensor domain. To deal with the weak fault characteristics extraction from multichannel signals under strong background noise, an adaptive low-rank tensor estimation model based on multichannel weak fault detection for bearings is investigated in this paper. The major contributions consist of the following three aspects.

(1) To tackle weak fault characteristics extraction from multichannel signals under strong background noise, an adaptive threshold function is introduced, and an adaptive low-rank tensor estimation model is constructed.
(2) A new sparsity metric-oriented parameter optimization strategy is provided for the adaptive low-rank tensor estimation model to further improve the estimation accuracy of weak fault characteristics extracted from multichannel signals.
(3) The effectiveness and superiority of the proposed method were verified through multichannel data from the repeatable simulation, the publicly available XJTU-SY whole lifetime datasets and an accelerated fatigue test of rolling bearings.

The structure of this paper is arranged as follows. Section 2 introduces the tensor-related theory and tensor characterization method. The proposed method is described in Section 3. In Section 4, the effectiveness and superiority of the proposed method are validated through multichannel simulation signals. The proposed method is also verified for its superiority through public bearing accelerated fatigue life datasets, and laboratory bearing accelerated fatigue test datasets in Section 5. Section 6 summarizes the main work and significance of this paper.

## 2. Methodology

### 2.1. Tensor Related Theory

Tensors are the high-dimensional extensions of vectors and matrices, symbolled as (A,B,C⋯) in this paper. The order of a tensor represents its dimension. The most commonly used tensor is the third-order tensor represented by the notation $A\in R^{n_{1}\times n_{2}\times n_{3}}$, where $n_{1},n_{2},n_{3}$ represent the size of each order. When the index of one dimension is fixed, the obtained subset matrices are called slices. One third-order tensor has three different slices, namely horizontal slices, lateral slices, and forward slices, represented sequentially as $A^{1}=A(h,:,:)$, $A^{2}=A(:,l,:)$ and $A^{3}=A(:,:,f)$.

**Figure 1:**
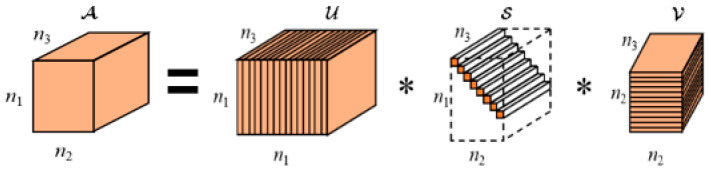
The diagram of TSVD.

Tensor singular value decomposition (TSVD) provides a simple and feasible mode decomposition approach for tensors [33]. For a third-order tensor $A\in R^{n_{1}\times n_{2}\times n_{3}}$, TSVD can decompose it into the form of tensor products by

(1) $$A=U*S*V^{T}$$

where $U\in R^{n_{1}\times n_{1}\times n_{3}}$ and $V\in R^{n_{2}\times n_{2}\times n_{3}}$ are orthogonal tensors and $S\in R^{n_{1}\times n_{2}\times n_{3}}$ is a diagonal tensor as shown in Figure 1. Through applying a Fourier transform along the 3rd-dimensional direction of the tensor, TSVD can be computed efficiently via multiple matrix SVD in the Fourier domain [29,37].

The *1* norm of a tensor is defined as the sum of absolute values of all elements in the tensor. The *Frobenius* norm of a tensor is defined as the square root of the sum of squares of all elements in the tensor. Based on the above TSVD theory, the tensor nuclear norm is defined as the sum of all singular values in the Fourier domain. For a third-order tensor $A\in R^{n_{1}\times n_{2}\times n_{3}}$, the corresponding calculation formulae are as follows.

(2) $${\left\|A\right\|}_{1}=\sum_{a,b,c}\left|A(a,b,c)\right|$$

(3) $${\left\|A\right\|}_{F}=\sqrt{\sum_{a,b,c}{A(a,b,c)}^{2}}$$

(4) $${\left\|A\right\|}_{*}=\sum_{i}\left(\sum_{j}{\hat{S}}^{\left(i\right)}\left(j,j\right)\right)$$

### 2.2. Tensor Construction of Multichannel Signals Based on Phase Space Reconstruction

The multichannel signal tensor in this paper will be constructed using the original time-series signal. The employment of the original time-series signal preserves the integrity of the raw data without losing the original information of the signal. Combined with the tensor, which is the most direct form of high-dimensional data expression, the high-order characterization of vibrational signals adopted in this paper can maximally retain the correlation information and potential data structure embedded within the original time-series data. In addition, according to chaos dynamics theory, the tensor characterization based on phase space reconstruction can better display the dynamic characteristics of the system and has been widely used in practice [38]. Therefore, the phase space reconstruction method is utilized in this section to tensorize the multichannel signals.

Assume that a *d*-channel signal $S=[s_{1}^{T}(t),\cdots ,s_{d}^{T}(t)]$ is acquired; every channel signal is $s_{i}(t)=[s_{i}(1),s_{i}(2),\cdots ,s_{i}(N)]$, i=1,⋯,d. Then, the observation tensor $Z\in R^{m\times L\times d}$ can be constructed based on phase space reconstruction as

(5) $$Z(:,:,i)=\left[\begin{array}{cccc} s_{i}(1) & s_{i}(2) & \cdots & s_{i}(L)\\ s_{i}(1+\tau ) & s_{i}(2+\tau ) & \cdots & s_{i}(L+\tau )\\ \vdots & \vdots & \ddots & \vdots \\ s_{i}(1+(m-1)\tau ) & s_{i}(2+(m-1)\tau ) & \cdots & s_{i}(N) \end{array}\right]$$

where *L* denotes the basic window length of the observation tensor, τ represents the time delay, *N* is the total length of signals, *m* denotes the embedding dimension, and *d* is the number of channels. Obviously, they satisfy as

(6) L=N−τ×(m−1).

**Figure 2:**
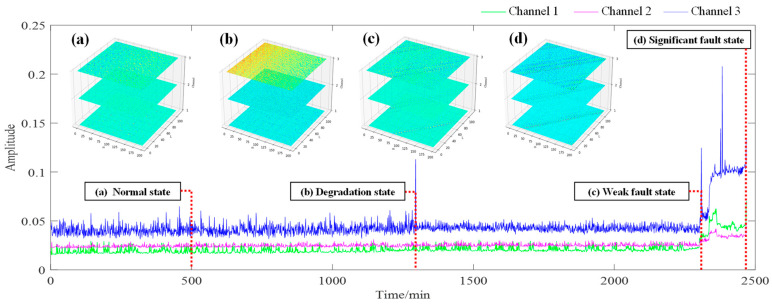
Whole lifetime root mean square (RMS) plot of a bearing.

Based on the characteristics of fault signals and expert knowledge, tensor construction of multichannel signals based on phase space reconstruction can effectively utilize the diversity of multichannel signals to enhance the representation of fault characteristics with reasonable structural parameters. For example, as shown in Figure 2, it is a whole lifetime root mean square (RMS) plot of a test bearing. In the figure, four representative states of the whole lifetime are selected and their multichannel signals are represented as observation tensors. Obviously, there are differences between multichannel signals, exhibiting diversity in both the time domain and tensor domain. At the same time, multichannel signals have consistency in characterizing the trend of fault development. And as the degree of fault increases, the fault characteristics reflect a certain structural correlation in multichannel tensors. Therefore, fault characteristics extraction based on multichannel signals has the advantages of data diversity and completeness. The tensor representation based on phase space reconstruction can effectively utilize the structural correlation between multichannel signals.

During the early bearing fault stage, the fault characteristics are very weak. Although multichannel signals contain richer fault characteristics, they are still submerged by strong background noise. How to utilize the structural characteristics of multichannel signals in the tensor domain to extract weak fault characteristic components is the key problem to tackle within this paper.

## 3. Proposed Method

### 3.1. The Low-Rank Property of Multichannel Fault Signals

**Figure 3:**
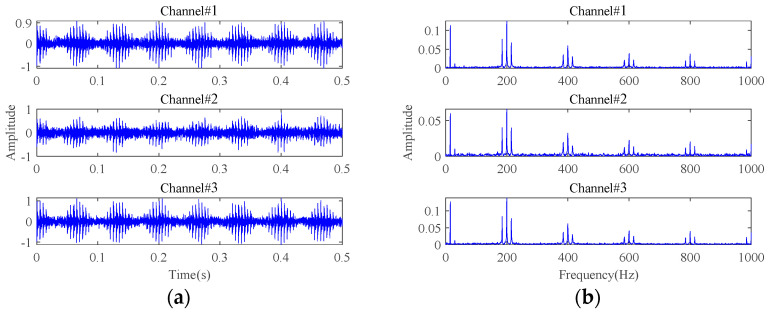
The simulated multichannel fault signals with an SNR of 20 dB: (**a**) time-domain waveforms; (**b**) frequency spectra.

**Figure 4:**
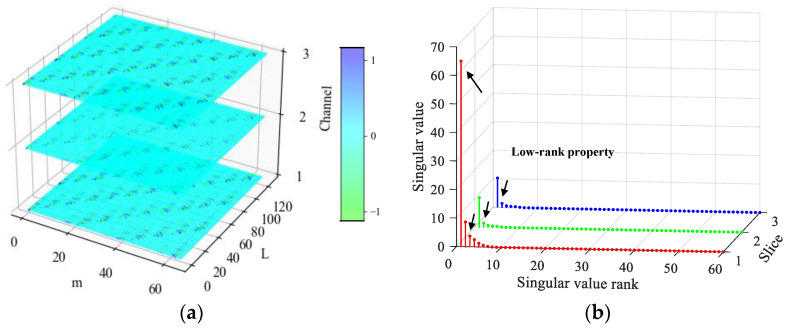
The simulated multichannel noise-free fault signals: (**a**) the observation tensor; (**b**) singular values of TSVD in the Fourier domain.

**Figure 5:**
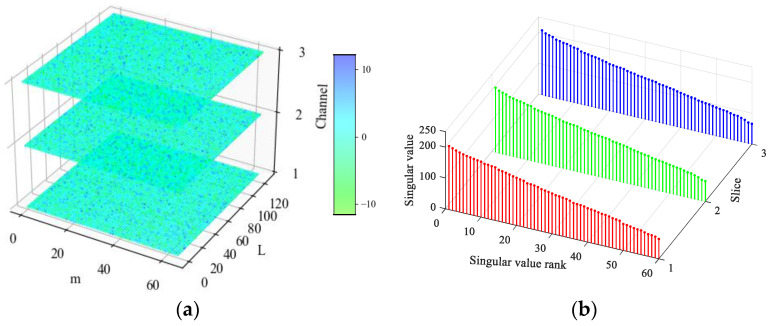
The simulated multichannel noisy signals with an SNR of −20 dB: (**a**) the observation tensor; (**b**) singular values of TSVD in the Fourier domain.

In this section, the simulated multichannel inner race fault signals of rolling bearings are used to reveal the low-rank property of multichannel fault signals in the tensor domain. The detailed simulation parameters are described in Section 4. To display the fault characteristics of multichannel signals, the bearing fault signals attached with Gaussian white noise are simulated at first, in which the signal-to-noise ratio (SNR) is set to 20 dB. The time-domain waveforms and frequency spectra of the simulated multichannel bearing fault signals are shown in Figure 3. Signals from the three channels have certain differences in the time domain and exhibit the same fault characteristics in the frequency domain. To find the influences of strong background noise on fault signals in the tensor domain, the noise-free fault signals and the noisy fault signals with an SNR of −20 dB were simulated. The simulated fault signals are transformed into the tensor domain through phase space reconstruction and then processed by TSVD. The results are shown in Figure 4 and Figure 5. It can be observed that the observation tensor of the noise-free fault signals exhibits a certain degree of low-rank structure in the high-dimensional domain, as shown in Figure 4a. And the singular values in each frontal slice obtained by TSVD mainly gather in the first few ranks in the Fourier domain. It can be concluded that the multichannel noise-free fault signals have a low-rank property in the tensor domain. However, for the noisy fault signals under strong background noise in Figure 5, the low-rank property of the fault signals cannot be observed anymore and is almost all masked by strong background noise in both the tensor domain and the Fourier domain.

Therefore, how to exploit the low-rank property of the multichannel fault signals in the tensor domain to achieve accurate extraction of weak fault characteristics under strong background noise is the key issue to tackle in this paper.

### 3.2. The Adaptive Low-Rank Tensor Estimation Model

For an observation tensor Z, assume that it consists of a fault characteristic component X and a noise component G. For the multichannel fault signals that have a low-rank property in the tensor domain, significantly different from random noise components, the fault characteristic component X can be roughly extracted from strong background noise by solving the following optimization problem.

(7) $$\underset{X,G}{\arg\min{\left\|X\right\|}_{*}}+\lambda {\left\|G\right\|}_{1}\;s.t.\;Z=X+G$$

where ${\left\|X\right\|}_{*}$ represents the tensor nuclear norm, and $\lambda {\left\|G\right\|}_{1}$ is a regularization term to balance the model against overfitting. The minimum nuclear norm constraint is an extremely strong convex constraint in mathematics, which can strongly induce a low-rank property of the estimated component X. Based on the tensor robust principal component analysis (TRPCA), Equation (7) can be transformed into the following low-rank tensor estimation model, relaxing the tensor nuclear norm constraints [29].

(8) $$\underset{X,G}{\min}\sum_{i=1}^{n_{3}}\sum_{j=1}^{n}\mathrm{soft}(\sigma_{j}^{\left(i\right)}(X),\gamma )+\lambda {\left\|G\right\|}_{1}\;s.t.\;Z=X+G$$

where soft(σ,γ)=max(σ−γ,0) represents the soft threshold function. The solution to Equation (8) can be obtained through the alternating direction method of multipliers (ADMMs) algorithm [39]. However, once a certain threshold is exceeded, the soft threshold function utilized in this model reduces all singular spaces equally at each iteration. This reduction strategy reduces the fault characteristic components significantly while weakening the noise, which is not conducive to weak fault characteristic extraction under strong background noise.

**Figure 6:**
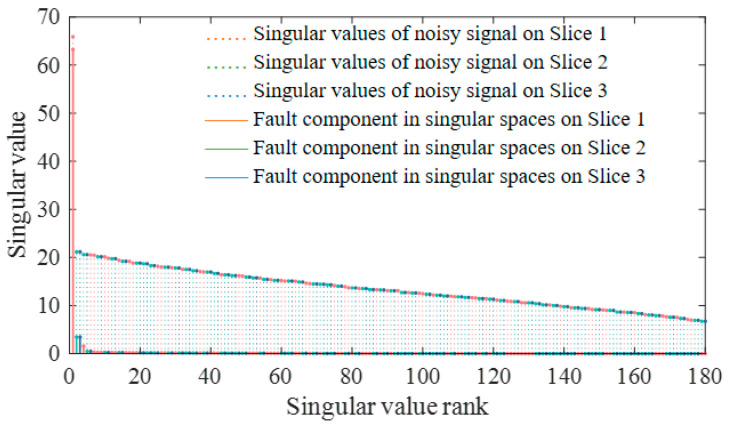
Noisy signal singular value distribution and projections of fault component.

It can be seen that singular values of the fault signals have significant low-rank properties in the Fourier domain in Figure 4. In order to clearly observe the amplitude proportion of the fault signal component in singular spaces, the noisy signals with an SNR of 5 dB are simulated, as an example. All singular values obtained by TSVD are arranged and displayed from largest to smallest, and the fault component is projected onto the corresponding singular spaces, as shown in Figure 6. It can be observed that the fault component is mainly distributed in the first few singular spaces, and as the singular value decreases, the proportion of the fault component decreases rapidly and is subsequently cut down to zero. Therefore, the threshold reduction strategies should be adaptively adjusted with singular values. In order to better fit the fault characteristic component, a more robust adaptive threshold function is introduced. Firstly, a threshold τ needs to be set. Secondly, an inversely proportional function is introduced to fit the association of singular values with reduction strategies. Thirdly, to ensure the non-convexity of the threshold function, a parameter γ is added to control the shape of the function. Finally, an adaptive threshold function is represented as

(9) $$f_{\tau ,\gamma }(\sigma )=\left\{ \begin{array}{l} 0\;\;\;\;\;\;\;\;\;\;\;\;\;\;\;\;\;\;\;\;\;\;\;\;\;,\;\;\sigma \leq \tau \\ \sigma \cdot \left(1-{\left(\frac{\tau }{\sigma }\right)}^{\gamma }\right)\;,\;\;\sigma >\tau \end{array}\right..$$

Combined with this adaptive threshold function, an improved model, adaptive low-rank tensor estimation model is constructed as follows.

(10) $$\underset{X,G}{\min}\sum_{i=1}^{n_{3}}\sum_{j=1}^{n}f_{\tau ,\gamma }(\sigma_{j}^{\left(i\right)}(X))+\lambda {\left\|G\right\|}_{1}\;s.t.\;Z=X+G$$

where $\sigma_{j}^{\left(i\right)}(X)$ is the *j*-th singular value in the *i*-th slice of tensor X in the Fourier domain.

### 3.3. MGISES-Oriented Parameter Optimization Strategy

To improve the fault characteristics extraction accuracy and maximize energy retention of fault signals, it is necessary to optimize the parameters τ and γ in the adaptive low-rank tensor estimation model. The fault of rolling bearings shows the impact characteristics in the vibration signal, which can be utilized to induce the parameter optimization strategy of the model for the accurate extraction of the fault characteristic components.

As an excellent sparsity metric, the Gini index (GI) is sensitive to the impact characteristics of signals and frequently used for fault detection in rotating mechanical equipment [40]. Compared with other metrics, GI has a unique resistance to random disturbances, which makes GI-based metrics less susceptible to random impulses caused by impacts on the outside of the bearing housing or electromagnetic interference [41]. Thus, GI is ideally suitable for weak fault detection in rotating mechanical equipment under strong background noise. Wang et al. proposed several GI-related sparsity metrics for machine condition monitoring [42,43,44].

Based on the advantages of GI and considering the requirement of simultaneous evaluation of multichannel signals, a new metric, multichannel GI of square envelope spectrum (MGISES), is designed in this paper for the evaluation of the sparsity of fault signals. For a vibration signal $s=(s_{1},\ldots ,s_{N})$, its square envelope spectrum can be obtained by $\mathrm{SES}=\mathrm{abs}(fft({\left|s\right|}^{2}))$. Then, the GI of the square envelope spectrum (GISES) can be defined as [45]

(11) $$\mathrm{GISES}=1-2\sum_{n=1}^{N}\frac{{\mathrm{SES}}_{\left(n\right)}}{{\left\|\mathrm{SES}\right\|}_{1}}(\frac{N-n+0.5}{N}).$$

For a *d*-channel signal $S=[s_{1}^{T}(t),s_{2}^{T}(t),\ldots ,s_{d}^{T}(t)]$, MGISES is defined as the average GISES of all channels as

(12) $$\mathrm{MGISES}=\frac{1}{d}\sum_{j=1}^{d}\mathrm{GISES}(s_{j}^{T}(t)).$$

**Figure 7:**
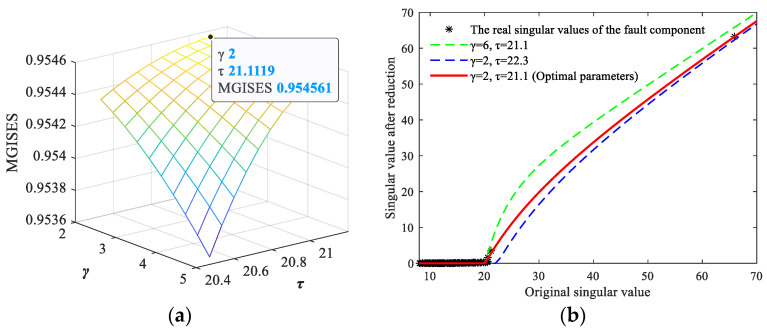
The MGISES-oriented parameter optimization: (**a**) the grid search process; (**b**) the optimized threshold function.

Then, the grid search algorithm [46] is used to search for the optimal parameters according to MGISES, and an MGISES-oriented parameter optimization strategy is developed. The detailed procedures of the MGISES-oriented parameter optimization are generalized in Algorithm 1. According to the low-rank property of the fault signal, the second largest singular value is chosen as the upper bound of the parameter τ. The parameter γ needs to be greater than one to ensure non-convexity, and its upper bound is chosen to be 10. The parameter optimization process and results of the simulated multichannel noisy signals in Figure 6 are displayed, as an example in Figure 7. It can be seen from Figure 7 that the threshold function obtained based on the proposed parameter optimization strategy excellently fits the real singular values of the fault component, maximizing signal energy retention of the fault characteristics under strong background noise. In order to demonstrate the influence of parameters on the threshold function, the functions with different parameters are also drawn in Figure 7b for comparison.
**Algorithm 1.** MGISES-oriented parameter optimization**Input:** $\gamma_{\min},\gamma_{\max},\tau_{\min},\tau_{\max}$1. Determine the resolution Δγ and Δτ
2. Calculate MGISES(*i, j*) for *i* = 1:*I*  for *j* = 1:*J*   $\gamma (i)=\gamma_{\min}+\Delta \gamma \times (i-1)$, $\tau (i)=\tau_{\min}+\Delta \tau \times (j-1)$   Calculate MGISES(*i, j*) according to γ(i) and τ(j)
   while $\gamma (i)\geq \gamma_{\max}\&\tau (j)\geq \tau_{\max}$
    break   end  end end**Output:** Parameters γ and τ with the maximum MGISES

### 3.4. Solution of Adaptive Low-Rank Tensor Estimation Model

In this section, the ADMM algorithm will be used for Equation (10). Firstly, the augmented Lagrangian function is constructed for Equation (10) as

(13) $$L_{\mu }(X,G,Y)=\sum_{i=1}^{n_{3}}\sum_{j=1}^{n}f_{\tau ,\gamma }(\sigma_{j}^{\left(i\right)}(\hat{X}))+\lambda {\left\|G\right\|}_{1}+\langle Y,X+G-Z\rangle +\frac{\mu }{2}{\left\|X+G-Z\right\|}_{F}^{2}$$

where Y represents the Lagrange multiplier, and μ denotes the penalty coefficient. According to the ADMM framework, the optimization of $L_{\mu }(X,G,Y)$ can be achieved iteratively by following the procedure:

(1) Update $X_{k+1}$*:* When fixing the variables $G_{k}$ and $Y_{k}$, the problem (13) can be simplified as

(14) $$\begin{array}{l} X_{k+1}=\underset{X}{\arg\min}\sum_{i=1}^{n_{3}}\sum_{j=1}^{n}f_{\tau ,\gamma }(\sigma_{j}^{\left(i\right)}(\hat{X}))+\langle Y_{k},X+G_{k}-Z\rangle +\frac{\mu }{2}{\left\|X+G_{k}-Z\right\|}_{F}^{2}\\ \;\;\;\;\;\;\;\;=\underset{X}{\arg\min}\sum_{i=1}^{n_{3}}\sum_{j=1}^{n}f_{\tau ,\gamma }(\sigma_{j}^{\left(i\right)}(\hat{X}))+\frac{\mu }{2}{\left\|X-(-G_{k}+Z-\frac{Y_{k}}{\mu })\right\|}_{F}^{2} \end{array}$$

(2) Update $G_{k+1}$*:* When fixing the variables $X_{k}$ and $Y_{k}$, the problem can be simplified as

(15) $$\begin{array}{l} G_{k+1}=\underset{G}{\arg\min}\;\lambda {\left\|G\right\|}_{1}+\langle Y_{k},X_{k+1}+G-Z\rangle +\frac{\mu }{2}{\left\|X_{k}+G-Z\right\|}_{F}^{2}\\ \;\;\;\;\;\;\;\;=\underset{G}{\arg\min}\;\lambda {\left\|G\right\|}_{1}+\frac{\mu }{2}{\left\|G-(-X_{k+1}+Z-\frac{Y_{k}}{\mu })\right\|}_{F}^{2} \end{array}$$

(3) Update $Y_{k+1}$*:* When fixing the variables $X_{k+1}$ and $G_{k+1}$, the problem can be simplified as

(16) $$Y_{k+1}=Y_{k}+\mu (X_{k+1}+G_{k+1}-Z)$$

Let M=Y/μ, the iteration process can be simplified to following three subproblems

(17) $$X_{k+1}=\underset{X}{\arg\min}\sum_{i=1}^{n_{3}}\sum_{j=1}^{n}f_{\tau ,\gamma }(\sigma_{j}^{\left(i\right)}(\hat{X}))+\frac{\mu }{2}{\left\|X-(-G_{k}+Z-M_{k})\right\|}_{F}^{2}$$

(18) $$G_{k+1}=\underset{G}{\arg\min}\;\lambda {\left\|G\right\|}_{1}+\frac{\mu }{2}{\left\|G-(-X_{k+1}+Z-M_{k})\right\|}_{F}^{2}$$

(19) $$M_{k+1}=M_{k}+X_{k+1}+G_{k+1}-Z$$

where ${\left(\cdot \right)}_{k}$ represents the *k*-th iteration.

**Figure 8:**
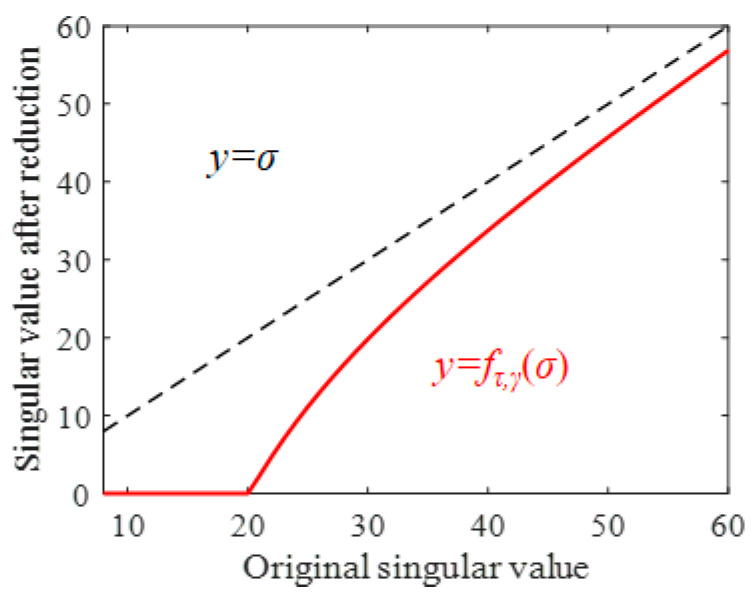
The illustration of the adaptive threshold function.

Problem (17) is the core procedure to achieve low-rank component extraction. Based on TSVD, Equation (17) can be decomposed into *n*_3_ subproblems as

(20) $$\begin{array}{l} {\hat{X}}_{k+1}^{\left(i\right)}=\underset{{\hat{X}}^{\left(i\right)}}{\arg\min}\sum_{j=1}^{n}f_{\tau ,\gamma }(\sigma_{j}^{\left(i\right)}(\hat{X}))+\frac{\mu }{2}{\left\|{\hat{X}}^{\left(i\right)}-(-{\hat{G}}_{k}^{\left(i\right)}+{\hat{Z}}^{\left(i\right)}-{\hat{M}}_{k}^{\left(i\right)})\right\|}_{F}^{2}\;\\ \;\;\;\;\;\;\;\;=\underset{{\hat{X}}^{\left(i\right)}}{\arg\min}\frac{\tau^{\gamma }}{\mu }\sum_{j=1}^{n}\frac{1}{{\left(\sigma_{j}^{\left(i\right)}(\hat{X})\right)}^{\gamma -1}}\sigma_{j}^{\left(i\right)}(\hat{X})+\frac{1}{2}{\left\|{\hat{X}}^{\left(i\right)}-(-{\hat{G}}_{k}^{\left(i\right)}+{\hat{Z}}^{\left(i\right)}-{\hat{M}}_{k}^{\left(i\right)})\right\|}_{F}^{2}\; \end{array}$$

which can be solved through

(21) $${\hat{X}}_{k+1}^{\left(i\right)}={\hat{D}}_{\tau ,\gamma }^{\left(i\right)}(-{\hat{G}}_{k}^{\left(i\right)}+{\hat{Z}}^{\left(i\right)}-{\hat{M}}_{k}^{\left(i\right)})$$

where ${\hat{D}}_{\tau ,\gamma }^{\left(i\right)}(\cdot )={\hat{U}}_{k}^{\left(i\right)}diag\{f_{\tau ,\gamma }(\sigma_{j}^{\left(i\right)}(\cdot ))\}{\hat{V}}_{k}^{(i)T}$, and 1≤j≤n is the singular value threshold algorithm [47]. The essence of this operation is to adaptively reduce the magnitude of all singular spaces at each iteration based on the proposed adaptive threshold function. As illustrated in Figure 8, the smaller singular values are rapidly reduced to zero. For the singular spaces containing fault components, the larger singular value is adaptively reduced according to its own magnitude. Therefore, the reduction strategy eliminates the noise component while avoiding a significant reduction in the useful singular space amplitude, which effectively realizes the energy retention of the low-rank fault component.

Based on the above derivation, the low-rank tensor estimation algorithm for solving Equation (10) is obtained. The detailed procedures of the algorithm are shown in Algorithm 2.
**Algorithm 2**. Solve Equation (10) by ADMM**Input:** tensor $Z\in R^{n_{1}\times n_{2}\times n_{3}}$, parameters λ,τ,γ,μ,ξ, maximum iterations N;**While** convergence is not satisfied **do**1. Update $X_{k+1}$ by: $X_{k+1}=\underset{X}{\arg\min}\sum_{i=1}^{n_{3}}\sum_{j=1}^{n}f_{\tau ,\gamma }(\sigma_{j}^{\left(i\right)}(\hat{X}))+\frac{\mu }{2}{\left\|X-(-G_{k}+Z-M_{k})\right\|}_{F}^{2}$; 2. Update $G_{k+1}$ by: $G_{k+1}=\underset{G}{\arg\min}\;\lambda {\left\|G\right\|}_{1}+\frac{\mu }{2}{\left\|G-(-X_{k+1}+Z-M_{k})\right\|}_{F}^{2}$;3. Update $M_{k+1}$ by: $M_{k+1}=M_{k}+X_{k+1}+G_{k+1}-Z$;4. Check for convergence: ${\left\|X_{k+1}-X_{k}\right\|}_{\infty }\leq \xi ,{\left\|G_{k+1}-G_{k}\right\|}_{\infty }\leq \xi ,{\left\|X_{k+1}+G_{k+1}-Z\right\|}_{\infty }\leq \xi$
**End****Output:** X

### 3.5. Weak Fault Detection Framework Based on Multichannel Signals

**Figure 9:**
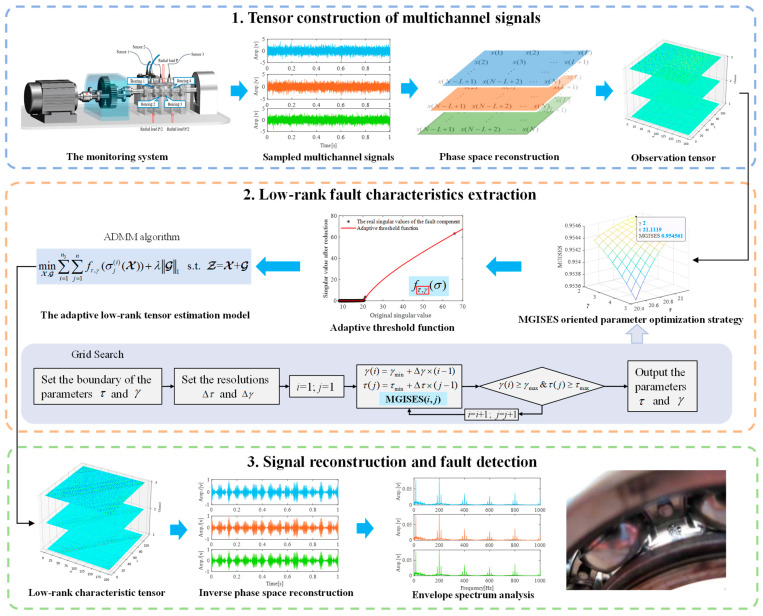
Flowchart of the proposed framework.

To tackle the weak fault characteristics of exact extraction from multichannel signals under strong background noise, an effective weak fault detection framework based on an adaptive low-rank tensor estimation model is proposed in this paper. The flowchart of the proposed framework is shown in Figure 9, which consists of the following three main procedures.

(1) Tensor construction of multichannel signals. Firstly, the multichannel signals of rolling bearings are sampled. Then, appropriate parameters of window length *L* and time delay τ are selected based on fault signal characteristics. Next, the multichannel signals are transformed into tensors based on phase space reconstruction. Then, the third-order observation tensor Z is constructed based on multichannel signals.

(2) Low-rank fault characteristics extraction. Two hyper-parameters in the proposed adaptive threshold function need to be optimized first. The MGISES-oriented parameter optimization strategy is utilized to obtain the optimal parameters based on the observation tensor Z. Then, an adaptive low-rank tensor estimation model can be constructed to estimate the low-rank component. Next, the ADMM algorithm is used to obtain the solution of this model. Finally, the low-rank tensor X representing the bearing fault characteristics can be obtained.

(3) Signal reconstruction and fault detection. The low-rank characteristic tensor X is reconstructed into multichannel signals by inverse phase space reconstruction. Envelope spectrum analysis can reflect the characteristics of vibration signals more comprehensively and intuitively. Therefore, the reconstructed multichannel signals are analyzed by envelope spectrum analysis to achieve weak fault detection at last.

## 4. Simulation Analysis

To verify the effectiveness of the adaptive low-rank tensor estimation method proposed in this paper, multichannel signals of rolling bearings with inner race fault and strong background noise are simulated and processed in this section. In addition, some comparative analysis is conducted on related algorithms as shown in Table 1. In order to verify the advantages of the adaptive threshold function, traditional low-rank tensor estimation is selected for comparison. To validate the effect of parameter optimization oriented by different metrics, we compared our method with the Kurtosis-oriented method and the Negative entropy-oriented method. To highlight the role that the tensor plays in the method of this paper, we compared our method with four advanced multichannel signal processing methods (i.e., MEMD, MVMD, MITD, and MSSD). MEMD and MVMD are multichannel mode decomposition methods proposed based on EMD and VMD, respectively, both of which can realize simultaneous decomposition of multi-dimensional signals. MITD has been pioneered for the adaptive processing of multi-loop data, which overcomes the problem of projection sensitivity. MSSD can extract adaptively multichannel mode components from multivariable signals containing different channels.

### 4.1. Weak Fault Detection Based on the Proposed Method

In engineering practice, the sampled multichannel vibration signals of rolling bearings not only contain fault impact components but also are affected by other rotating connections and external noise. At the same time, there are certain differences between channels. Therefore, 3-channel signals of bearings can be expressed as [36]

(22) S=MY+N

where Y represents the simulated signal consisting of three source signals, N denotes the strong background noise, and M is a 3 × 3 random matrix.

Y in (22) is composed of three source signals as

(23) $$Y=[y_{1}^{T}(t),y_{2}^{T}(t),y_{3}^{T}(t)]$$

(24) $$\left\{ \begin{array}{l} y_{1}(t)=p(t)\sum_{i=0}^{I}m_{i}(t)\cos(2\pi f_{e}t-n_{i}(t)+\varphi_{i})\\ \;\;\;\;\;\;\;\;\;m_{i}(t)=E_{i}e^{-\zeta (t-iT-\eta_{i})}u(t-iT_{c}-\eta_{i})\\ \;\;\;\;\;\;\;\;\;n_{i}(t)=\sum_{j=1}^{J}M_{ij}\sin(2\pi ft+\varphi_{ij}) \end{array}\right.$$

(25) $$y_{2}\left(t\right)=0.01\cos\left(2\pi f_{2}t+20\right)$$

(26) $$y_{3}\left(t\right)=0.02\sin\left(2\pi f_{3}t+10\right).$$

The fault signal of rolling bearings, recorded as $y_{1}(t)$ in Equation (24), can be viewed as a superimposition of several pulses [48,49]. The functions $m_{i}(t)$ and $n_{i}(t)$ are modulation components corresponding to amplitude and frequency, respectively. The function p(t) refers to the modulation effect generated by the transmission from the fault location to the sensor mounting location. For inner race fault, $p(t)=P[1+cos(2\pi f_{r}t)]$. $E_{i}$,$M_{ij}$ and P are amplitudes, which in this case take the values 0.25, 1, and 3, respectively. $T_{c}$ is the period of transients. In this paper, the bearing inner race fault signal is simulated with 10,010 sampling points and 10 kHz sampling frequency, respectively. The settings of the other parameters are shown in Table 2. The functions $y_{2}(t)$ and $y_{3}(t)$ are two low-amplitude harmonic interfering signals with the characteristic frequency of *f*_2_ = 20 Hz and *f*_3_ = 50 Hz, respectively.

Then, a 3-channel signal Y can be simulated by a random mixing matrix M, as follows

(27) $$M=\left[\begin{array}{ccc} 0.7820 & 0.4191 & 0.3906\\ 0.4904 & 0.1158 & 0.2848\\ 0.8571 & 0.3243 & 0.1519 \end{array}\right].$$

**Figure 10:**
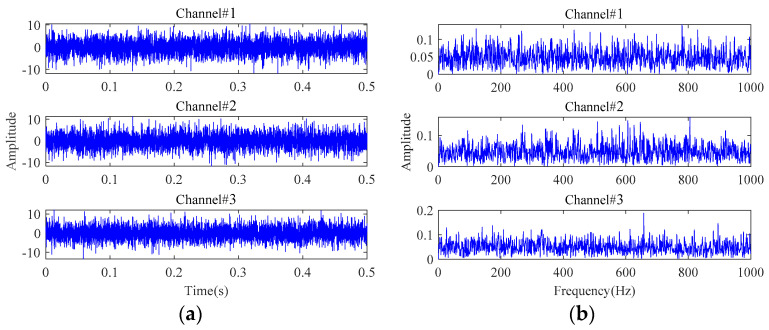
The simulated multichannel fault signals with an SNR of −10 dB: (**a**) time-domain waveforms; (**b**) frequency spectra.

**Figure 11:**
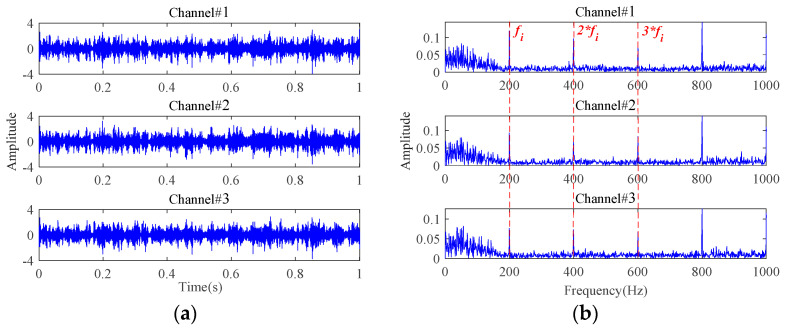
The results of the proposed method for signals with an SNR of −10 dB: (**a**) time-domain waveforms; (**b**) envelope spectra.

Attached with Gaussian white noise $N(N=\{n_{i}(t)\},i=1,2,3)$, the time-domain waveforms and frequency spectra of the simulated multichannel signals with an SNR of −10 dB are plotted in Figure 10. It can be observed that the fault characteristic frequencies are completely masked, rendering any useful fault-related information unreadable. In order to exactly extract the bearing fault characteristics under strong background noise, the proposed weak fault characteristics extraction method is applied to this simulated multichannel noisy signals. The results are displayed in Figure 11. The inner race fault frequency and its harmonic components can be clearly seen from the extracted signals characterizing the inner race fault, and the interference of the background noise has been effectively removed.

### 4.2. Comparative Analysis

**Figure 12:**
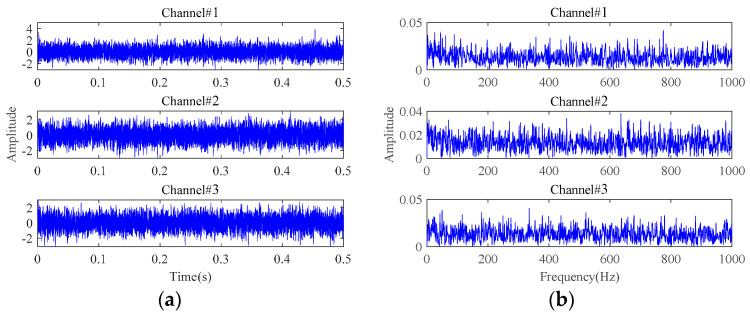
The results of **Method 1** for signals with an SNR of −10 dB: (**a**) time-domain waveforms; (**b**) envelope spectra.

The results of fault characteristics extraction based on **Method 1** are shown in Figure 12. Almost no useful information about the fault characteristics can be observed from time-domain waveforms and envelope spectra. Therefore, **Method 1** cannot achieve effective fault characteristics extraction under strong background noise. Different from the adaptive low-rank tensor estimation model, **Method 1** utilizes the soft threshold function to reduce the noisy components during each iteration optimization. Once the singular values are bigger than a certain threshold, the reduction in singular spaces remains equal, independent of the singular values. When processing signals with strong background noise, this procedure undoubtedly would lead to a synchronous reduction in the characteristic components in the dominant singular spaces, leading to poor fault characteristics extraction performance. By comparing the results in Figure 11 and Figure 12, the superiority of the proposed adaptive threshold function and adaptive low-rank tensor estimation model in dealing with strong background noise can be further verified.

**Figure 13:**
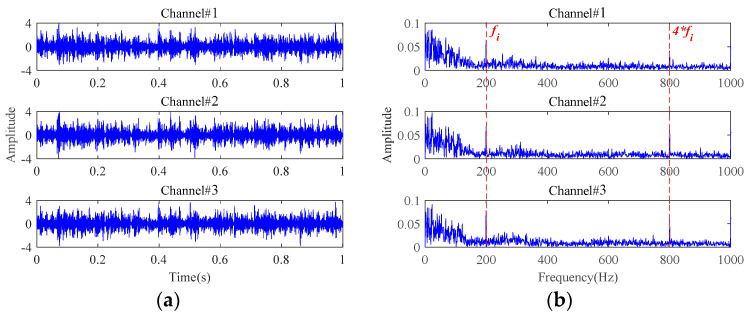
The results of **Method 2** for signals with an SNR of −10 dB: (**a**) time-domain waveforms; (**b**) envelope spectra.

**Figure 14:**
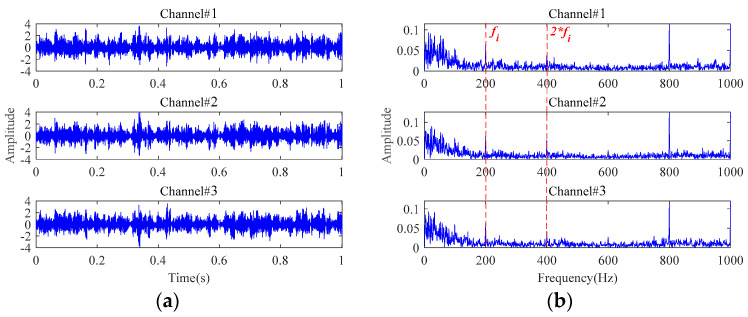
The results of **Method 3** for signals with an SNR of −10 dB: (**a**) time-domain waveforms; (**b**) envelope spectra.

The results of fault characteristics extraction based on **Method 2** and **Method 3** are shown in Figure 13 and Figure 14, respectively. Obviously, **Method 2** and **Method 3** can extract some fault characteristics related components, which is attributed to the low-rank tensor estimation model proposed in this paper. However, their effectiveness is significantly inferior to the proposed method in Figure 11. This is due to the fact that the proposed MGISES has a unique resistance to random disturbances and is less susceptible to extreme values or outliers, so the proposed method is less susceptible to random impulses caused by impacts on the outside of the bearing housing or electromagnetic interference. In contrast, the two sparsity metrics, Kurtosis and Negative entropycrag, which are used as comparison methods, have no such effect. Therefore, the oriented parameter optimization strategy is more helpful for weak fault characteristics extraction of the proposed method under strong background noise.

**Figure 15:**
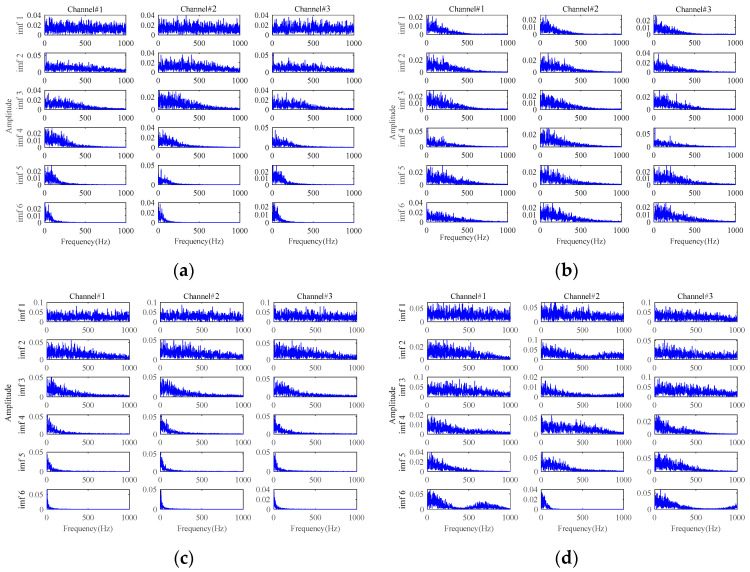
The results for signals with an SNR of −10 dB based on: (**a**) **Method 4**; (**b**) **Method 5**; (**c**) **Method 6**; (**d**) **Method 7**.

The results based on the other four methods (i.e., **Method 4**, **Method 5**, **Method 6,** and **Method 7**) are displayed in Figure 15, by selecting the appropriate number of decomposition layers and intrinsic mode functions (IMFs), respectively. From the results of these four comparison methods in Figure 15, only the results of **Method 5** vaguely show fault-related information. There is almost no fault-related information in the results of the other three methods. Both of them fail to realize useful noise reduction for signals with strong background noise. For these four methods, not considering the inter-channel correlation, they cannot fully exploit the correlation information between multichannels to realize the noise reduction. This will result in information redundancy in multichannel signals, causing interference in fault characteristics extraction. Instead, the proposed method is tensor-based, which fully utilizes the structural correlation between the multichannel signals. It is thus verified once again that the tensor-based characteristics extraction method is able to achieve better noise reduction results.

## 5. Experiment Verification

Early faults are usually very weak and masked by strong surrounding background noise. Therefore, it is difficult to detect. Timely fault detection in the early fault stage could undoubtedly provide more time and possibility, for preventive maintenance of equipment, more reference basis for further life prediction, and effectively reduce maintenance costs and machine downtime. The proposed method is applied for early fault detection in real scenarios to further validate its superiority in multichannel weak fault characteristics extraction under strong background noise. Two different bearing whole lifetime datasets, the publicly available XJTU-SY bearing whole lifetime dataset from Xi’an Jiaotong University [50] and the rolling element bearing dataset of an accelerated fatigue test, are utilized for validation.

### 5.1. Case 1: XJTU-SY Bearing Lifetime Dataset

**Figure 16:**
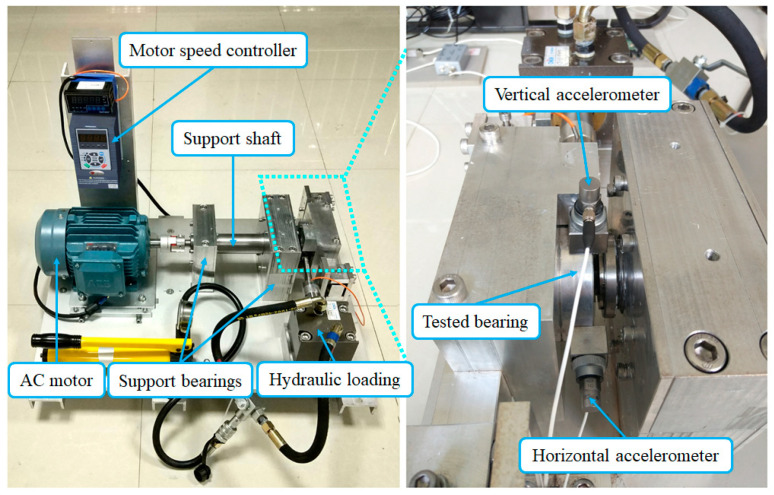
Bearing testing platform of XJTU-SY.

The bearing accelerated fatigue testing platform of XJTU-SY is shown in Figure 16. The testbed consists of an AC induction motor, hydraulic loading system, motor speed controller, support bearings, and support shaft. The type of tested bearing is an LDK UER204. Datasets of Bearing1_1 and Bearing1_3 are selected for further analysis, whose rotating speed and radial force are 2100 rpm and 12 kN, respectively. The sampling frequency is 25.6 kHz. A total of 1.28 s of data were recorded per minute. Two accelerometers were installed to collect the vibration signals through two channels.

**Figure 17:**
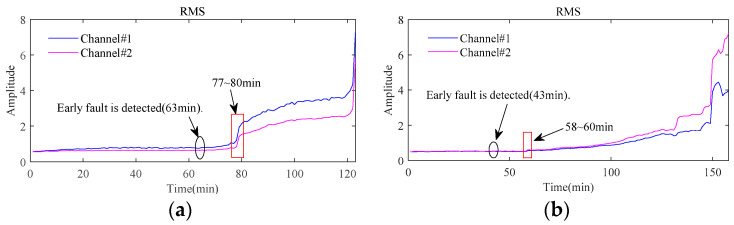
Root mean square of bearings in the whole lifetime: (**a**) Bearing1_1; (**b**) Bearing1_3.

**Figure 18:**
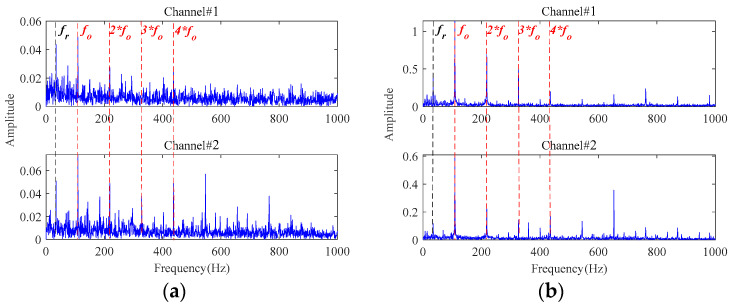
The envelope spectra of the sampled signals: (**a**) at 81 min in Bearing1_1; (**b**) at 61 min in Bearing1_3.

Figure 17 displays the root mean square (RMS) of the vibration signals throughout the whole lifetime for both bearings. A distinct rise in the RMS value, especially when followed by a continuous increase, usually indicates the occurrence of fault for bearings as highlighted by the red rectangular box in Figure 17. The signals sampled around these time points are further analyzed to study the state of the bearings. The envelope spectra of the signal at 81 min in Bearing1_1 and 61 min in Bearing1_3 are displayed in Figure 18, from which the rotating frequency and the characteristic frequency of bearing outer race fault can be clearly observed in both channels from these two bearings. It indicates that a local fault has occurred in the outer race during the degradation processes.

Due to the low sensitivity of the RMS metrics to early faults, more detailed fault information cannot be obtained from the RMS plots. Instead, the proposed method is capable of exactly extracting early weak fault characteristics that are submerged by the strong background noise. To detect the early fault as quickly as possible, the signals sampled before the obvious fault stage are analyzed using the proposed method.

**Figure 19:**
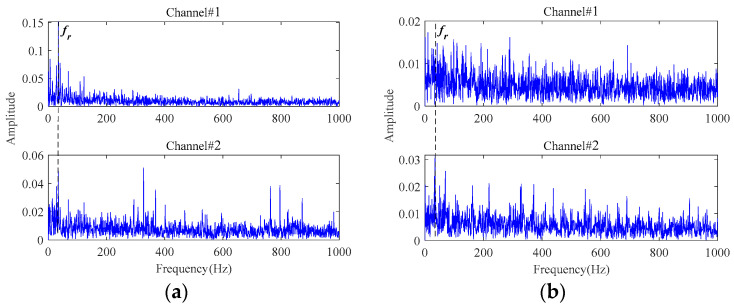
The envelope spectra of the sampled signals: (**a**) at 63 min in Bearing1_1; (**b**) at 43 min in Bearing1_3.

**Figure 20:**
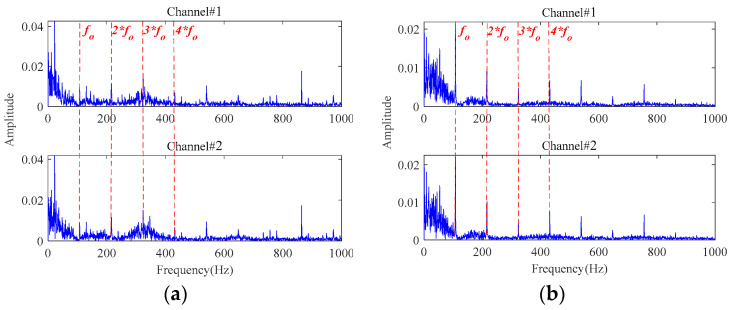
The envelope spectra of the denoised signals by the proposed method: (**a**) at 63 min in Bearing1_1; (**b**) at 43 min in Bearing1_3.

Firstly, a third-order tensor with the size of 138 × 245 × 2 is constructed by multichannel signals. Then, the observation tensor is processed through the MGISES-oriented parameter optimization strategy, the weak fault characteristics extraction by the adaptive low-rank tensor estimation model, inverse phase space reconstruction, and envelope spectrum analysis of the denoised signals. The early faults can be detected at 63 min in Bearing1_1 and 43 min in Bearing1_3 at the earliest. The envelope spectra of the original signals are displayed in Figure 19. It is only possible to observe the rotating frequency of the bearings, while almost all fault characteristic frequencies of both bearings and their harmonics are drowned out by the strong background noise. Then, the envelope spectra of the finally extracted fault characteristic signals are shown in Figure 20. It is observed that the fault characteristic frequencies of both bearings and their harmonics can be distinctly identified after noise reduction by the proposed method. It indicates that these two bearings start to undergo a weak degradation phenomenon at this point, producing early faults. After this moment, both bearings gradually began to show obvious faults. Finally, the testing bearings run to failure. Then, it was found through disassembly and inspection that these two bearings did indeed have outer race faults. Therefore, the early faults of both bearings can be clearly and timely identified based on the proposed method.

With the aim of validating the superiority of the “very early” detection of the proposed method, the earliest time points at which faults can be clearly identified are also analyzed by the proposed method for Bearing2_1 and Bearing3_1. The results are listed in Table 3. Furthermore, early fault detection results from the relevant references are also listed in Table 3 for comparison. It is obvious that the proposed method has a distinct advantage in early fault detection.

**Figure 21:**
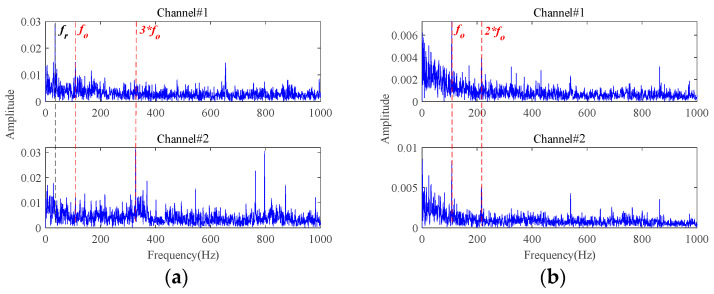
The envelope spectra of the denoised signals based on **Method 1**: (**a**) at 63 min in Bearing1_1; (**b**) at 43 min in Bearing1_3.

Then, the signals sampled at 63 min in Bearing1_1 and 43 min in Bearing1_3 are also processed by the methods in Table 1 for comparative analysis. Figure 21 shows the envelope spectra of the denoised signals by **Method 1**. From Figure 21a, it can be found that only the fault characteristic frequency in Channel#1 can be observed in the extracted signal for Bearing1_1. In Figure 21b, the fault characteristic frequency and two times the fault characteristic frequency can be seen in both channels for Bearing1_3. However, obvious background noise is still visible in the envelope spectra of both channel signals.

**Figure 22:**
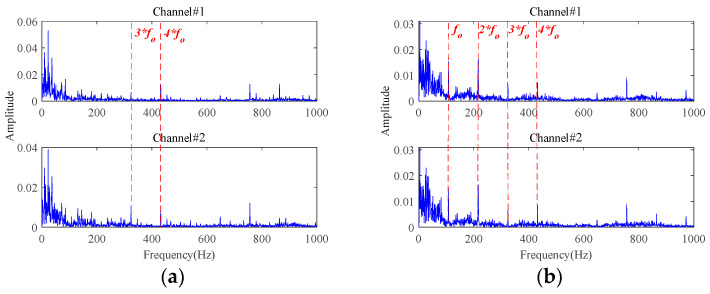
The envelope spectra of the denoised signals based on **Method 2**: (**a**) at 63 min in Bearing1_1; (**b**) at 43 min in Bearing1_3.

**Figure 23:**
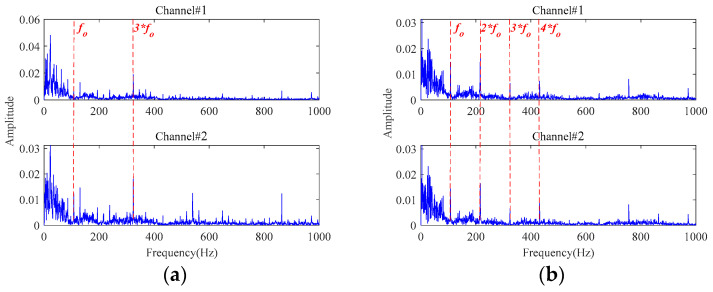
The envelope spectra of the denoised signals based on **Method 3**: (**a**) at 63 min in Bearing1_1; (**b**) at 43 min in Bearing1_3.

Figure 22 and Figure 23 show the envelope spectra of the denoised signals by **Method 2** and **Method 3**. The fault characteristic frequency and its harmonic frequencies are clearly seen in the signals denoised by **Method 2** and **Method 3**, which are attributed to the utilization of the fault characteristics extraction method based on the adaptive threshold function and the adaptive low-rank tensor estimation model. However, compared to Figure 20, their fault characteristics extraction capabilities, as well as their energy retention effects, are relatively poor.

**Figure 24:**
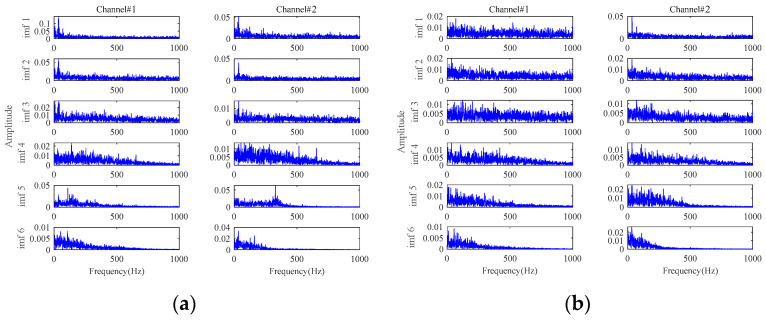
The envelope spectra of the denoised signals based on **Method 4**: (**a**) at 63 min in Bearing1_1; (**b**) at 43 min in Bearing1_3.

**Figure 25:**
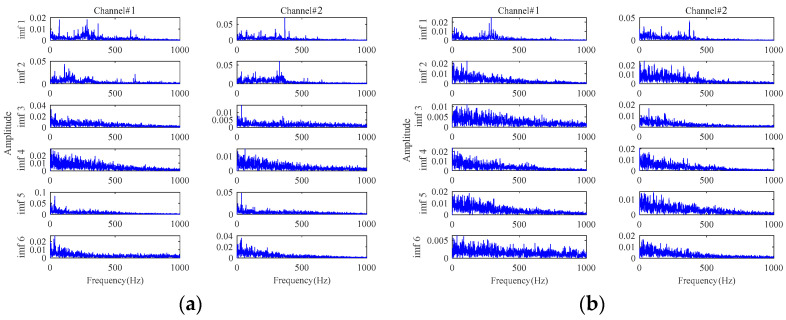
The envelope spectra of the denoised signals based on **Method 5**: (**a**) at 63 min in Bearing1_1; (**b**) at 43 min in Bearing1_3.

**Figure 26:**
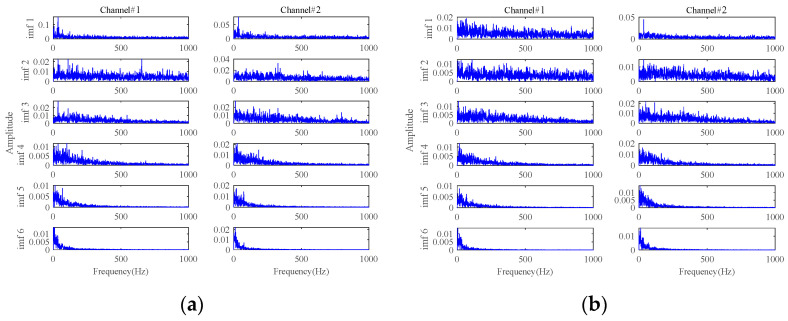
The envelope spectra of the denoised signals based on **Method 6**: (**a**) at 63 min in Bearing1_1; (**b**) at 43 min in Bearing1_3.

**Figure 27:**
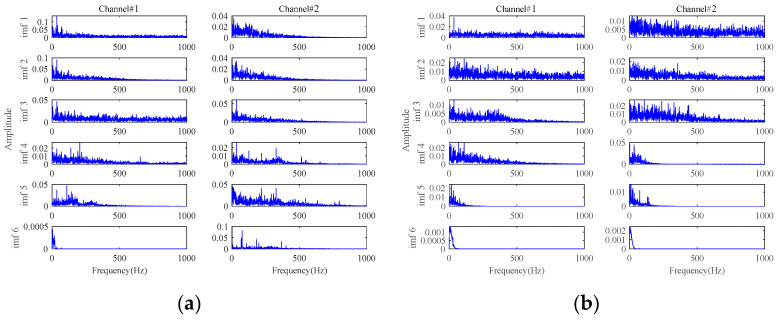
The envelope spectra of the denoised signals based on **Method 7**: (**a**) at 63 min in Bearing1_1; (**b**) at 43 min in Bearing1_3.

Figure 24, Figure 25, Figure 26 and Figure 27 show the envelope spectra of the denoised signals of the two bearings by the other four methods in Table 1 (i.e., **Method 4**, **Method 5**, **Method 6,** and **Method 7**) through selecting the appropriate number of decomposition layers and intrinsic mode functions (IMFs). The best noise reduction was achieved by **Method 7**. The results for the two bearings show clear fault frequencies but still suffer from noise interference. The remaining three methods can see little fault-related information in the result plots. Compared to Figure 20, these four methods are generally unable to effectively extract early weak fault characteristics from strong background noise.

The proposed method can effectively utilize the structural correlation between multichannel signals to enhance the low-rank characteristics extraction ability. At the same time, under the guidance of the sparsity metric, it can maintain superior fault characteristics extraction ability in the presence of strong background noise. Therefore, for the “very early” fault stage, the proposed method achieves much better performances compared with the other four methods.

### 5.2. Case 2: An Accelerated Fatigue Test of Rolling Element Bearings

**Figure 28:**
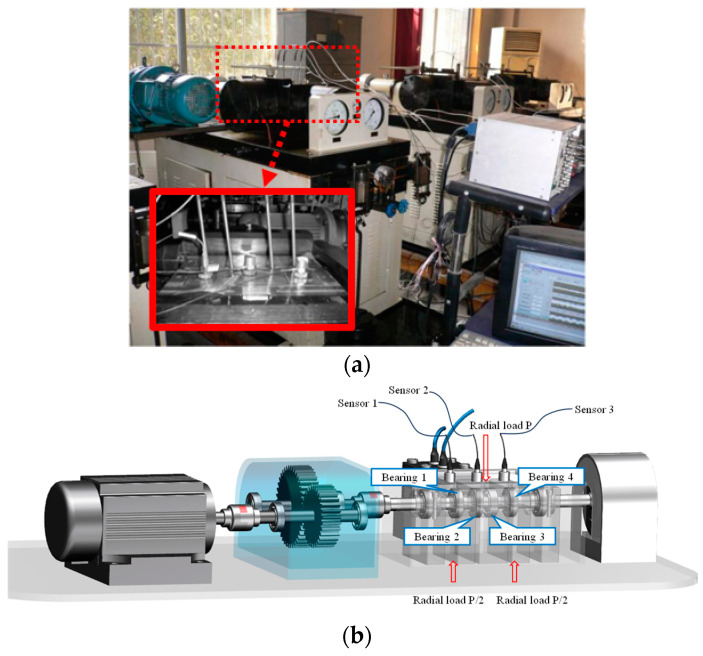
The accelerated bearing life tester (ABLT-1A): (**a**) physical drawing of the test bench; (**b**) schematic diagram of the test bench structure.

To further verify the superiority of the proposed method in multichannel weak fault characteristic extraction and early fault detection, an accelerated fatigue test was conducted on the accelerated bearing life tester (ABLT-1A), as shown in Figure 28a. The experimental platform consisted of a lubrication system, transmission system, data acquisition system, AC motor, and loading system. Four bearings typed 6307 were synchronously installed at four locations for the accelerated life test. To accelerate the bearing fatigue process, a radial load of 12.744 kN was applied. The data acquisition system of the test bench included three PCB348A acceleration sensors to collect vibration signals generated by four bearings. The position of the sensors and the load are shown in Figure 28b. The type of data acquisition card was NI PCI-6023E. The sampling frequency was 25.6 kHz, and 0.8 s of data were recorded per minute. The corresponding parameters of the testing bearings are provided in Table 4. During the test, the rotating speed of the shaft was 3000 rpm. The fault characteristic frequencies of the bearings are calculated, as shown in Table 5.

**Figure 29:**
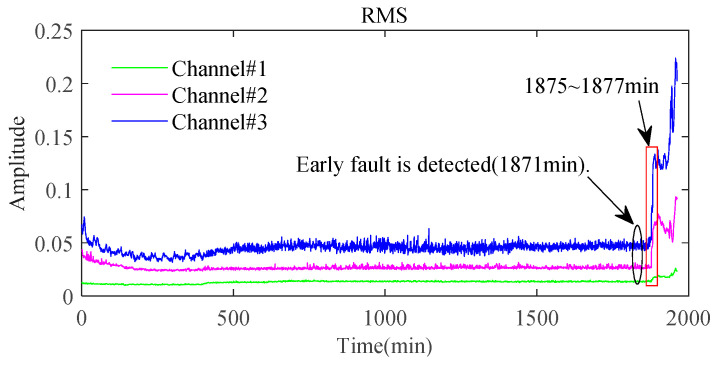
RMS of bearing in the whole lifetime.

**Figure 30:**
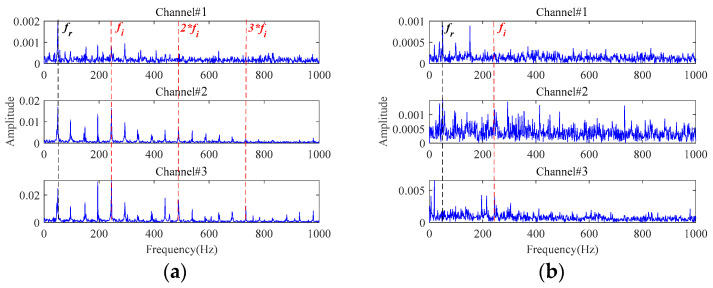
The envelope spectra of the sampled signals: (**a**) at 1878 min; (**b**) at 1871 min.

Furthermore, the signals sampled from the three channels contain significant differences. As seen from Figure 29, the signal from Channel#3 is more sensitive to the degradation process and contains larger fluctuations caused by noise interference compared with the other two channels. However, it has been found that signals from Channel#2 and Channel#3 contain much clearer fault frequency characteristics from Figure 30a. For signals at 1871 min in Figure 30b, the amplitude at the rotating frequency is more pronounced in Channel#1. However, peaks at the fault characteristic frequency only can be visualized from the other two channels, although they are masked under strong interference noise. Therefore, multichannel signals contain richer fault characteristic information and can effectively assist the fault characteristics extraction under strong background noise.

**Figure 31:**
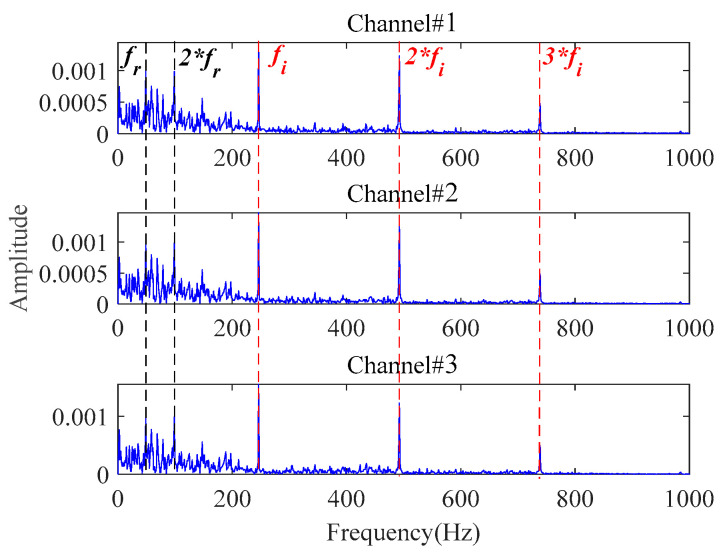
The envelope spectra of the final extracted fault signals based on the proposed method at 1871 min.

**Figure 32:**
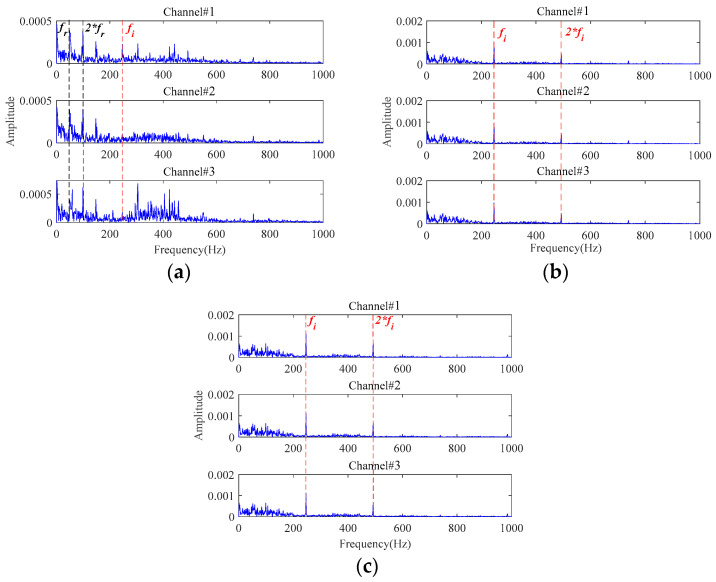
The envelope spectra of the final extracted fault signals based on: (**a**) **Method 1**; (**b**) **Method 2**; (**c**) **Method 3**.

**Figure 33:**
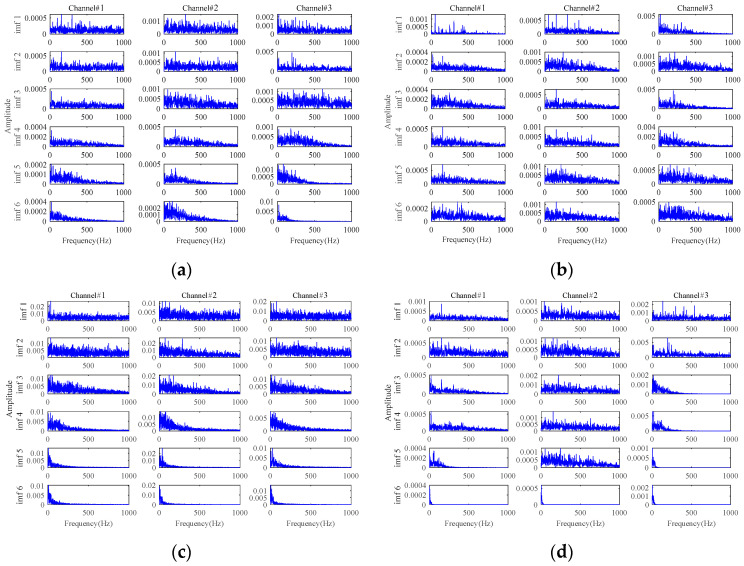
The envelope spectra of the final extracted fault signals based on: (**a**) **Method 4**; (**b**) **Method 5**; (**c**) **Method 6**; (**d**) **Method 7**.

Then, the 3-channel signals sampled at 1871 min are processed by the proposed method to extract the early fault characteristics. The size of the constructed observation tensor size is 196 × 114 × 3. Processed by the proposed method, the envelope spectra of the final extracted fault signals are shown in Figure 31. To verify the superiority of the proposed method, methods in Table 1 are also utilized to process these multichannel signals. The final results are displayed in Figure 32 and Figure 33.

In Figure 31, the rotating frequency and its harmonics can be identified in the low-frequency range. More importantly, significant inner race fault characteristic frequencies and their harmonics can be distinctly observed without any background interference. It indicates that the bearing starts to undergo a weak degradation phenomenon at this point, producing an early fault. Therefore, the early fault can be detected at 1871 min with the proposed method. Then, an early warning can be provided before the fault worsens. Figure 30a confirms that a distinct inner race fault indeed occurs in the later stage of the bearing lifetime. The proposed method achieves satisfying results on the early fault characteristics extraction in the presence of strong background noise in this application.

From the results of the comparative methods in Figure 32a, **Method 1** has a relatively poor ability for fault characteristics extraction. As shown in Figure 32a, the fault characteristics of the inner race fault are relatively obvious in Channel#1. However, the fault characteristic frequency cannot be clearly observed in the other two channels. There are still obvious interference components in the extracted signals. The envelope spectra of the denoised signal by **Method 2** and **Method 3** are shown in Figure 32b,c, and it can be found that there are obvious fault characteristic frequencies in all three channels. But the amplitudes of the fault characteristic frequency in the extracted signals are much smaller than that in Figure 31, which validates the important role of the proposed MGISES-oriented parameter optimization strategy in energy retention. The results of the other four methods in Table 1 (i.e., **Method 4**, **Method 5**, **Method 6,** and **Method 7**) are shown in Figure 33. The results of **Method 4** and **Method 6** have fault-related information, but there is a large amount of noise, which fails to achieve good noise reduction. The results of **Method 5** and **Method 7** have obvious fault-related information. However, they fail to maintain the energy of the fault characteristics, and the noise still affects the extraction results.

In conclusion, the proposed method has achieved excellent performance in multichannel weak fault characteristics extraction under strong background noise. With the advantage of multichannel synchronous processing based on tensor decomposition, it can effectively remove the interference of strong background noise. In addition, it can better preserve the energy of fault characteristic components while removing strong background noise interference.

## 6. Conclusions

In this study, a weak fault detection framework is proposed as an effective method for multichannel signals under strong background noise. Firstly, the multichannel signals are transformed into a tensor by phase space reconstruction, which has obvious advantages in characterizing fault information for multichannel signals. And the low-rank property of multichannel fault signals in the tensor domain is revealed. Secondly, an adaptive threshold function is formulated according to the singular value distribution of the fault component and an adaptive low-rank tensor estimation model is constructed for weak fault characteristics extraction from multichannel signals under strong background noise. Thirdly, a new sparsity metric-oriented parameter optimization strategy is provided for the adaptive low-rank tensor estimation model to further improve the accurate estimation of weak fault characteristics from multichannel signals. Finally, an effective weak fault detection framework based on an adaptive low-rank tensor estimation model is formed for multichannel signals under strong background noise.

It is demonstrated by simulated multichannel signals and experimental analysis that the proposed method exhibits much better performance than other multichannel signal processing methods. First, the proposed method is compared with the traditional tensor estimation method. Results verify the superiority of the proposed adaptive threshold function and adaptive low-rank tensor estimation model in dealing with strong background noise. Second, compared to the Kurtosis/Negative entropy-oriented adaptive low-rank tensor estimation method, the proposed MGISES-oriented adaptive low-rank tensor estimation method is more helpful for weak fault characteristics extraction under strong background noise. Third, compared to advanced multichannel signal analysis methods (i.e., MEMD, MVMD, MITD, and MSSD), the proposed method verifies once again that the tensor-based characteristics extraction method is able to achieve better fault characteristics extraction results. In summary, this work can provide a reference for the research of multichannel/multisensory signal processing in the Industry 4.0 era. However, this method is based on a sole low-rank assumption, which will encounter difficulties in handling compound fault situations. In the future, to enrich and accelerate the development of intelligent diagnosis for compound fault, more in-depth studies for mechanical equipment not confined to bearings (e.g., gears, shafts, and other rotating parts) will be conducted on multichannel signal processing and feature extraction.

## Figures and Tables

**Table 1:** Comparative algorithms.

Method	Algorithms Used	Key Points of Comparisons
**Method 1**	Traditional low-rank tensor estimation method [29]	Threshold function
**Method 2**	Kurtosis-oriented adaptive low-rank tensor estimation method	Parameter optimization strategy
**Method 3**	Negative entropy-oriented adaptive low-rank tensor estimation method	
**Method 4**	MEMD [20]	Tensor based method
**Method 5**	MVMD [21]	
**Method 6**	MITD [24]	
**Method 7**	MSSD [25]	

**Table 2:** Parameters of the fault signal.

$\varphi_{i}$	$\varphi_{ij}$	$f_{e}$	ζ	$\eta_{i}$	$f_{r}$	f
0	0	2000 Hz	800	0.02/*f*	15 Hz	200 Hz

**Table 3:** Comparisons of early fault detection.

Bearing Dataset	Whole Lifetime	Detected Early Fault Points	Results of Proposed Method
Bearing1_1	123 min	67 min [11]	**63 min**
Bearing1_3	158 min	59 min [51]	**43 min**
Bearing2_1	491 min	453 min [52]	**434 min**
Bearing3_1	2538 min	2368 min [53]	**2321 min**

**Table 4:** Corresponding parameters of the testing bearings.

Type	Pitch Diameter	Ball Diameter	Ball Number	Contact Angle (°)
6307	58.5 (mm)	13.494 (mm)	8	0

**Table 5:** The fault characteristic frequencies of bearing 6307 (Hz).

Type	*f_r_*	*f_i_*	*f_o_*	*f_b_*	*f_c_*
6307	50	246	153	102	19

## Data Availability

Data is unavailable due to privacy.
